# Equilibrium studies of cobalt(II) extraction with 2-pyridineketoxime from mixed sulphate/chloride solution

**DOI:** 10.1007/s10967-015-4246-7

**Published:** 2015-06-20

**Authors:** Karolina Wieszczycka, Marta Krupa, Aleksandra Wojciechowska, Irmina Wojciechowska, Andrzej Olszanowski

**Affiliations:** Institute of Chemical Technology and Engineering, Poznan University of Technology, ul. Berdychowo 4, 60-965 Poznan, Poland

**Keywords:** Pyridylketoxime, Cobalt(II) extraction, Cobalt(II) speciation, Chloride/sulphate leaching, Chloride/sulphate solution

## Abstract

In present paper the equilibrium of cobalt extraction with 1-(2-pyridyl)tridecan-1-one oxime from the chloride/sulphate solutions was studied. The presented results indicated that extraction depends on a number of process variables, including the pH, metal and Cl^−^ concentration in the aqueous feed, and concentration of the oxime in the organic phase. The created cobalt-complexes with the 2-pyridine ketoxime were stable and only concentrated HCl was found to be a suitable stripping agent for coordinated metal. The separation of Co(II) from Zn(II), Ni(II) and Cu(II) was also studied, but the selective recovery of the metals was possible using the multi-stage stripping process.

## Introduction

Cobalt is a strategic and critical metal, which is used in many branches of industry e.g. as a component in superalloys, as a binder for tungsten carbide cutting tools, as pigments in glass, ceramics and paints, as catalysts in the petroleum industry and as an additive to Ni-metal hydride battery [1]. Those unique properties insure that the demand for cobalt will grow, therefore, its efficient recovery from poor primary sources is the most important goal to achieve.

Chloride leaching process using HCl is one of the most effective recovery route of cobalt from ore, concentrates and other primary sources, but, an optimisation of cobalt recovery, which related with a process selectivity and its less environmental influences led to develop the less aggressive leaching systems based on chloride salts or chloride/sulphate mixture [2–4]. Hybrid hydrochloric–sulphuric acidic leaching of a laterite ore has been proposed by Intec Limited and in this concept H_2_SO_4_ was used to regenerate HCl—a main leaching agent. Unfortunately, this process was not as effective as the chloride-circuits, but the observed effects led to continue of the studies [4]. A different hybrid process operates in a mixed chloride–sulphate magnesium brine system has been proposed by Anglo Research Nickel process (ARNi process) [5, 6]. In this concept decoupling reagents’ regeneration is promoted by utilising lowered solubility of magnesium sulphate in magnesium chloride solution and as a consequence, the resulting hydrochloric acid acts as the main circulating leach agent. Cominco Engineering Services Limited has also proposed chloride–sulphate leaching process, but the recovery of metals such as nickel, copper and cobalt from ore has been investigated using an acidic solution containing halide and sulphate ions in the presence of oxygen [7].

The pregnant solutions obtained in the chloride–sulphate leaching processes should be treated to selective separation of all dissolved payable metals in further stages. Solvent extraction is a process in which valuable metals are separated from worthless or other valuable metals, however, in case of cobalt extraction a lot of researchers have focused on an extraction and separation of cobalt from other metals from sulphate, ammonia or chloride solutions. In case of the mixed sulphate/chloride solutions, only NaPC-88A was studied as a potential selective extractant of cobalt(II) and nickel(II), but the selectivity of the process depended on the pH and the extractant’s concentration [8].

Over the last few years the extraction of metals ions from different aqueous solutions by hydrophobic 2-, 3- and 4-pyridylketoximes has been widely investigated. Especially, oxime of 1-(2-pyridyl)tridecan-1-one has shown good extraction abilities towards all studied metals copper(II), zinc(II), cadmium(II) and iron(III) ions from chloride and chloride/ammonia solutions [9–15], as well as copper(II) and nickel(II) ions from solutions containing both chloride and sulphate salts [16, 17]. Recent studies have also indicated the possibility to apply the oxime of 1-(2-pyridyl)tridecan-1-one for a separation of cobalt from nickel sulphate solution [18], but so far, the most efficient extraction of cobalt(II) ions using this oxime has been achieved only from the chloride/nitrate solution [19]. It has been assumed that the addition of chloride ions to cobalt sulphate solution should improve the extraction efficiency by oxime of 1-(2-pyridyl)tridecan-1-one, and also change the complexation mechanism. Therefore, in the present work, an extraction of cobalt(II) ions from the aqueous feed solutions containing both chloride and sulphate ions by oxime of 1-(2-pyridyl)tridecan-1-one was selected for a detailed analysis.

## Experimental

### Reagents

All reagents used in this study were of a reagent grade. Toluene (≥99.5 %, POCH, Poland) and decan-1-ol (≥98 %, Merck, Germany) were used as components of the organic phase. Sodium chloride (p.a., Chempur, Poland), hydrochloric acid (37 %) (p.a., POCH, Poland), sulphuric acid (p.a., POCH, Poland), cobalt(II) sulphate (heptahydrate) (p.a., Sigma-Aldrich, Germany) were used to compose the aqueous phase.

**Fig. 1:**
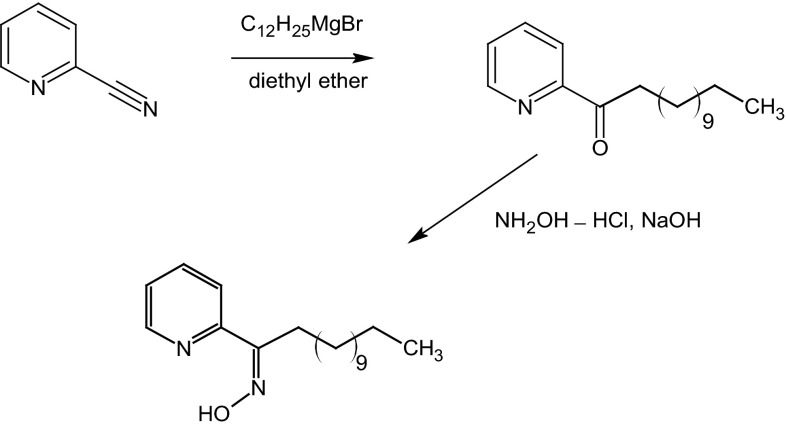
Synthesis of 1-(2-pyridyl)tridecan-1-one oxime

The oxime of 1-(2-pyridyl)tridecan-1-one (Fig. 1) was synthesised according procedure described in previous paper [9]: ^1^H NMR (400 MHz; CDCl_3_) *δ* (ppm): 9.53 (s, 1H, OH); 8.62 [m, 1H, H_py_(6)]; 7.83 [dd, 1H, H_py_(3), *J* **=** 11 and 7.8 Hz]; 7.69 [td, 1H, H_py_(4), *J* **=** 7.6 and 1.6 Hz]; 7.29 [ddd, 1H, H_py_(5), *J* **=** 1.1, 4.8 and 7.4 Hz]; 2.99 (t, 2H, CH_2_); 1.58 (q, 2H, CH_2_); 1.27–1.29 (m, 18H, CH_2_); 0.90 (t, 3H, CH_3_); ^13^C NMR (100 MHz; CDCl_3_) *δ* (ppm): 160.7; 154.0; 149.0; 136.3; 123.5; 120.9; 32.8; 31.9; 29.9; 29.7; 29.6; 29.4; 29.3; 29.1; 26.2; 25.6; 22.7; 14.1; LC-ESI/MS m/z (%): 291.2 (100) (MH)^+^; 274.0 (6.9); 256.2 (2.8); 235.4 (2.2); 179.0 (7.3); 161.2 (9.7); 151 (5.3); 133 (4.9); 79.2 (3.47); 52.2 (6.9).

### Extraction procedure

Basic extraction and stripping studies were carried out in a test tube using organic to aqueous phases volume ratio (O: A) equal to 1. Both phases were shaken at room temperature (21–23 °C) using an orbital shaker Bio-mix BWR 04 and then separated in a separating funnel. Each experiment was carried out at least in duplicates, and the results agreed within 3–4 %. Aqueous feed solutions were prepared by dissolving the appropriate amounts of CoSO_4_ (heptahydrate, Sigma-Aldrich, Germany), Na_2_SO_4_ (POCH, Poland) and NaCl (POCH, Poland) in ultrapure water. The pH of aqueous solutions was adjusted to a desired value by adding 2 % H_2_SO_4_ (POCH, Poland) solution using combined glass pH electrode DG111-SC (T50 Titrator, Mettler Toledo). Toluene (Sigma-Aldrich, Germany) with 10 % (v/v) addition of decan-1-ol (Sigma-Aldrich, Germany) was used as an organic diluents [toluene with 10 % (v/v) decan-1-ol were chose because, they were not creating emulsion. This opportunity was used to faster separation two phases]. All reagents used in this study were of reagent purity grade.

### Calculation

The metal content in the organic phase was determined by a mass balance between the concentration of the metal in the aqueous phase before and after the extraction. Distribution coefficient *D* was calculated from the following equation:

1 $$ D = \frac{{[{\text{Co}}]_{\text{org}} }}{{[{\text{Co}}]_{\text{aq}} }} $$

where [Co]_aq_ and [Co]_org_ are the cobalt(II) ions concentration after the extraction in the aqueous and organic phases. The estimation of cobalt complexes composition in an organic phase, equilibrium constants of the extraction, as well as a speciation analysis of cobalt(II) in an aqueous chloride/sulphate solution required a calculation of ions activity $a_{i}$.

2 $$ a_{i} = c_{i} \gamma_{i} $$

where *c*_*i*_ and *γ*_*i*_ are the concentration and activity coefficient of the positive or negative ionic species, respectively.

The activity coefficients of each ion in the aqueous phase were calculated using the Bromley equation [20], which can be adopted to concentrated solutions (up to *I* = 6 mol/dm^3^):

3 $$ { \log }_{\gamma } = - \frac{{0.5108\left| z \right|^{2} I^{0.5}  }}{{1 + I^{0.5} }} +  F_{i} $$

where, for specific cation *c* and anion *a*, the term *F*_*i*_ becomes:

4 $$ F_{c} = \mathop \sum \limits_{a} \left[ {\frac{{\left( {0.06 + 0.6 B_{ca} } \right)\left| {z_{c} z_{a} } \right|}}{{\left( {1 + \frac{1.5}{{\left| {z_{c} z_{a} } \right|}}I} \right)^{2} }} + B_{ca}  } \right] Z_{ca}^{2} m_{a} $$

5 $$ F_{a} = \mathop \sum \limits_{c} \left[ {\frac{{\left( {0.06 + 0.6 B_{ca} } \right)\left| {z_{c} z_{a} } \right|}}{{\left( {1 + \frac{1.5}{{\left| {z_{c} z_{a} } \right|}}I} \right)^{2} }} + B_{ca}  } \right]Z_{ca}^{2} m_{c} $$

6 $$ Z_{ca} = \frac{{\left| {z_{c} } \right| + \left| {z_{a} } \right|}}{2} $$

where *z*_*c*_ and *z*_*a*_ represent cation and anion charge number, respectively. *B*_*ca*_ represents the Bromley constant.

## Result and discussions

### Effect of shaking time

The effect of shaking time on the extraction of 0.01 mol/dm^3^ Co(II) from aqueous sulphate/chloride solution (0.5 mol/dm^3^${\text{SO}}_{4}^{2-}$ and 1 mol/dm^3^ Cl^−^) using 0.1 mol/dm^3^ 1-(2-pyridyl)tridecan-1-one oxime dissolved in toluene with 10 % (v/v) addition of decan-1-ol was investigated over the range 5–60 min. The results have clearly indicated that equilibration was reached after 10 min, but the 15 min shaking was selected to further studies.

### Effect of pH

**Fig. 2:**
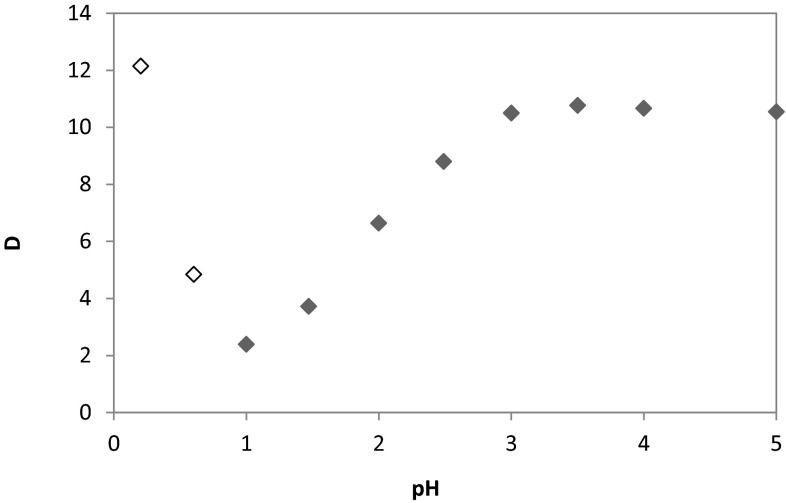
Influence of pH on cobalt(II) distribution coefficient ([Co^+2^] **=** 0.01 mol/dm^3^; [${\text{SO}}_{4}^{2-}$] **=** 0.5 mol/dm^3^; [Cl^−^] = 1 mol/dm^3^; [oxime] = 0.1 mol/dm^3^; organic diluent: toluene with 10 % (v/v) addition of decan-1-ol; ◊-emulsion was observed)

The effect of pH on the extraction of 0.01 mol/dm^3^ of Co(II) ions from chloride/sulphate aqueous solutions (0.5 mol/dm^3^${\text{SO}}_{4}^{2-}$ and 1 mol/dm^3^ Cl^−^) using 0.1 mol/dm^3^ 1-(2-pyridyl)tridecan-1-one oxime dissolved in toluene with 10 % (v/v) addition of decan-1-ol was studied over the pH range 0.25–5.5. Presented results have shown a linear dependence of the cobalt extraction and the pH of the aqueous feed (Fig. 2). This dependence is very clear in the range of pH 1–3, wherein the extraction increases linear from 71 to 91 %. Further increase of the pH does not affect the extraction and the efficiency is approximately 91–92 %. Very good extraction results are also observed at strong acidic range of the pH (below 1), but unfortunately during the metal extraction an emulsion is observed.

### Effect of chloride ions concentration

**Fig. 3:**
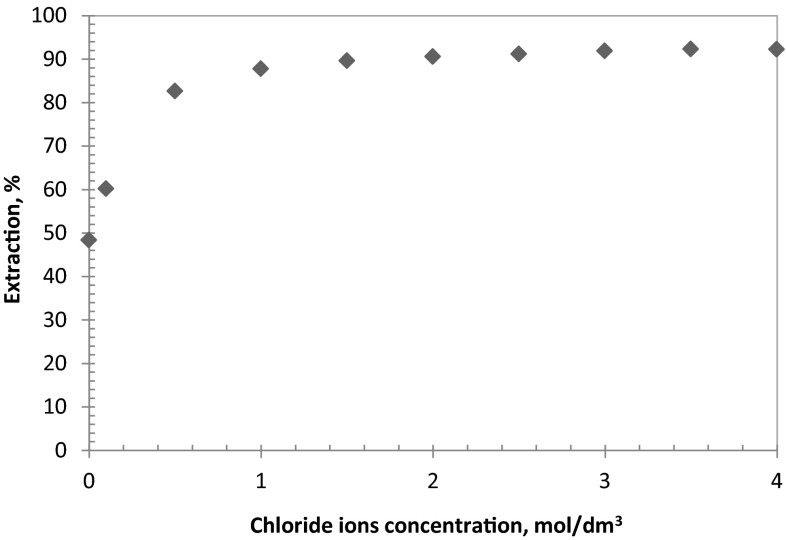
Influence of chloride ions concentration on cobalt(II) extraction {[Co^+2^] = 0.01 mol/dm^3^; [${\text{SO}}_{4}^{2-}$] = 0.5 mol/dm^3^; pH 5; [oxime] = 0.1 mol/dm^3^; organic diluent: toluene with 10 % (v/v) addition of decan-1-ol}

**Fig. 4:**
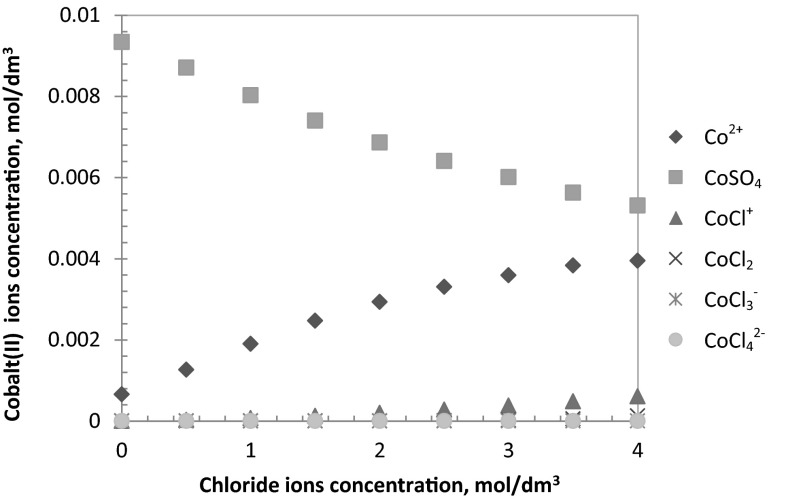
Speciation diagram of cobalt(II) species in chloride-sulphate solutions {[Co^2+^] = 0.01 mol/dm^3^; [${\text{SO}}_{4}^{2-}$] = 0.5 mol/dm^3^; pH = 5}

The effect of chloride ions concentration was estimated for the aqueous solutions containing a variable chloride ions’ concentration (from 0 to 4 mol/dm^3^) and at a constant sulphate ions concentration (0.5 mol/dm^3^). The pH of the aqueous feed solutions was equal to 3.5 and 5.0. The extraction results show that, regardless the aqueous phase’s pH, the influence of the chloride ions concentration on cobalt ions’ extraction is the most visible at the initial range of Cl^−^ concentration (example at pH 3.5 the extraction increases from 50 % for 0 mol/dm^3^ Cl^−^ to 89 % for 1 mol/dm^3^ Cl^−^) and above 1 mol/dm^3^ Cl^−^ the extraction stabilises enabling the metal recovery on the level of 90–92 % (Fig. 3). This relationships can be attributed to dominating cobalt-chloride species coordination, however, a diagram distribution of all of cobalt(II) species dissolved in chloride/sulphate aqueous feed eliminates this theory (Fig. 4). It has been found that cobalt(II) sulphate dominates, depending on the composition of aqueous solution, but the increasing concentration of chloride ions decreases cobalt(II) sulphate fraction in favour of mainly Co^2+^.

### Effect of extractant concentration

The effect of the oxime concentration on the extraction of 0.01 mol/dm^3^ Co(II) from aqueous sulphate/chloride solution (0.5 mol/dm^3^${\text{SO}}_{4}^{2-}$ and 1 mol/dm^3^ Cl^−^; pH = 0.5, 3.5 and 5) was studied with the varying 1-(2-pyridyl)tridecan-1-one oxime concentrations (0.01–0.2 mol/dm^3^).

**Fig. 5:**
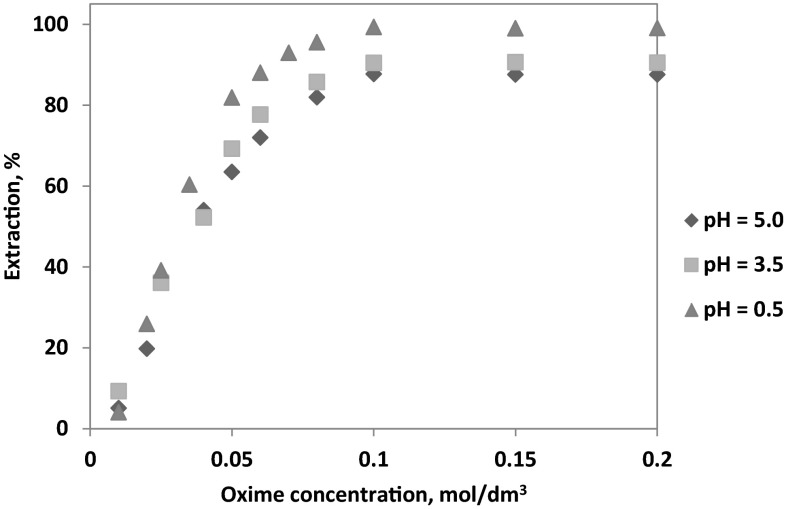
Influence of 1-(2-pyridyl)tridecan-1-one oxime concentration on extraction of Co(II) ions at different pH {[Co^2+^] **=** 0.01 mol/dm^3^; [${\text{SO}}_{4}^{2-}$] **=** 0.5 mol/dm^3^; [Cl^−^] **=** 1 mol/dm^3^; organic diluent: toluene with 10 % (v/v) addition of decan-1-ol}

During the extraction using the organic solutions containing from 0.01 to 0.05 mol/dm^3^ of the oxime, it has been observed that the extraction of cobalt(II) ions linear increases from 9.3 to 69.2 at pH 3.5 or from 5.1 to 63.4 % at pH 5 (Fig. 5). Further increase of the oxime concentration to a level above 0.08 mol/dm^3^ results of a maximum recovery of metal, which mainly depends on the pH of the aqueous phase: 99–93 % extraction of cobalt(II) ions at pH of 0.5, 87.5–90.4 % extraction at pH of 3.5 and 87.7–90.5 % extraction at pH of 5.

### Effect of temperature

**Fig. 6:**
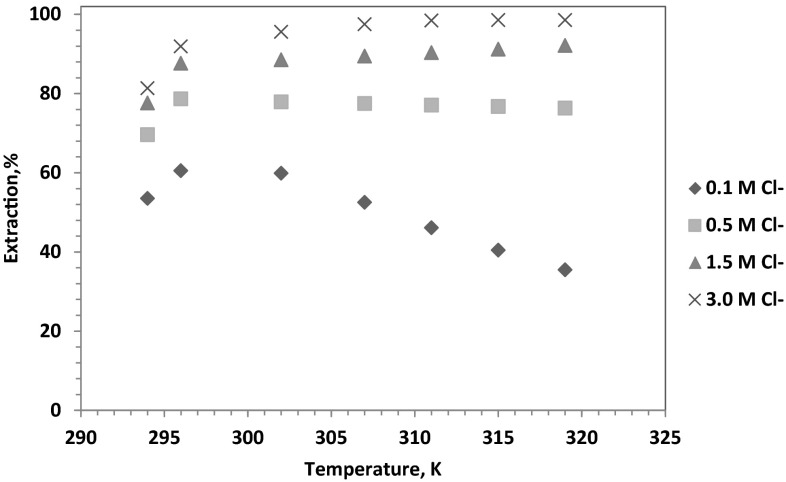
Influence of temperature on extracion of Co(II) ions with 1-(2-pyridyl)tridecan-1-one oxime dissolved in toluene with 10 % (v/v) addition of decan-1-ol {[Co^2+^] = 0.01 mol/dm^3^; [${\text{SO}}_{4}^{2-}$] = 0.5 mol/dm^3^; [Cl^−^] = 0.1, 0.5, 1.5 and 3 mol/dm^3^}

The influence of temperature on extraction of cobalt(II) ions from solutions containing 0.5 mol/dm^3^${\text{SO}}_{4}^{2-}$ and different concentration of chloride ions (0.01–4 mol/dm^3^ Cl^−^) with 1-(2-pyridyl)tridecan-1-one oxime dissolved in toluene with 10 % addition of decan-1-ol was studied in the temperature range of 294–319 K. The experimental results showed that the extraction percentages were closely related to the composition of the aqueous phase (Fig. 6). It was observed that in case of the solutions containing 0.5 mol/dm^3^${\text{SO}}_{4}^{2-}$ and 0.5 mol/dm^3^ Cl^−^ with increasing temperature the extraction initially increased from 69.6 (294 K) to 78.6 % (296 K) and then slightly decreased to value 76.3 % (319 K), but in case of the solution containing 0.5 mol/dm^3^${\text{SO}}_{4}^{2-}$ and 3 mol/dm^3^ Cl^−^ the increasing temperature increased the extraction from 81.3 (299 K) to 98.9 % (319 K). The enthalpy of the cobalt extraction were also calculated and the results obtained from the slopes of the Van’t Hoff plots [21] indicated the endothermic nature of the extraction process from the solution containing 0.5 mol/dm^3^${\text{SO}}_{4}^{2-}$ and 1.5 or 3 mol/dm^3^ Cl^−^ (∆*H* = 17.23 and 108.14 kJ/mol, respectively), whereas the cobalt(II) extraction from solution containing 0.5 mol/dm^3^${\text{SO}}_{4}^{2-}$ and 0.1 mol/dm^3^ Cl^−^ was exothermic process (∆*H* = −41.13 kJ/mol).

### Effect of cobalt(II) ions concentration

The effect of cobalt(II) ions concentration on the extraction was studied over the range of Co^2+^ 0.01–0.15 mol/dm^3^ at constant concentration sulphate ions (0.5 mol/dm^3^) and constant concentration chloride ions (1 or 3 mol/dm^3^). The pH of the aqueous feed solutions was equal to 5. The toluene with 10 % (v/v) addition of decan-1-ol of 1-(2-pyridyl)tridecan-1-one oxime (0.1 mol/dm^3^) was used as the organic phase.

**Fig. 7:**
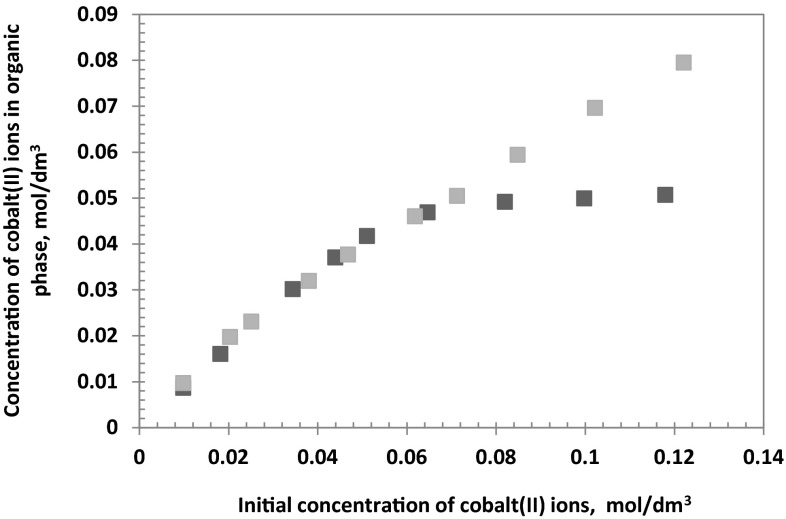
Relation between initial cobalt ions concentration in aqueous phase and concentration of cobalt ions in organic phase after extraction {[Co^2+^] = 0.01 mol/dm^3^; [${\text{SO}}_{4}^{2-}$] **=** 0.5 mol/dm^3^; [Cl^−^] = 1 (* Filled black square*) or 3 (* Filled grey square*) mol/dm^3^; pH 5; organic diluent: toluene with 10 % (v/v) addition of decan-1-ol}

The obtained data presented in Fig. 7, have shown that in case of the aqueous solutions containing 1 mol/dm^3^ Cl^−^, the amount of extracted cobalt(II) ions increases from 0.0088 to 0.0380 mol/dm^3^ along with the increase of initial cobalt(II) ions concentration from 0.010 to 0.044 mol/dm^3^. The shaking of the organic phase with the aqueous solutions containing Co^2+^ above 0.05 mol/dm^3^ results in an achieving of a maximum loading capacity of 0.048–0.050 mol/dm^3^.

A different relation has been observed for the aqueous feed solutions containing besides sulphate and cobalt(II) ions, 3 mol/dm^3^ Cl^−^ (Fig. 7). It has been observed that in all studied range of cobalt(II) ions concentration, the amount of the extracted metal ions increases linearly even for the solution containing more than 0.1 mol/dm^3^ Co^2+^. This effect may be attributed to the coordination of cobalt-chloride species, which have a tendency to aggregation especially at higher concentrations of chloride solutions [19]. The molecular aggregate formed an association complex. Finally, this effect enables to achieve a higher oxime capacity 0.078 mol/dm^3^ Co(II) [0.78 mol of Co(II) per mol of the oxime].

**Fig. 8:**
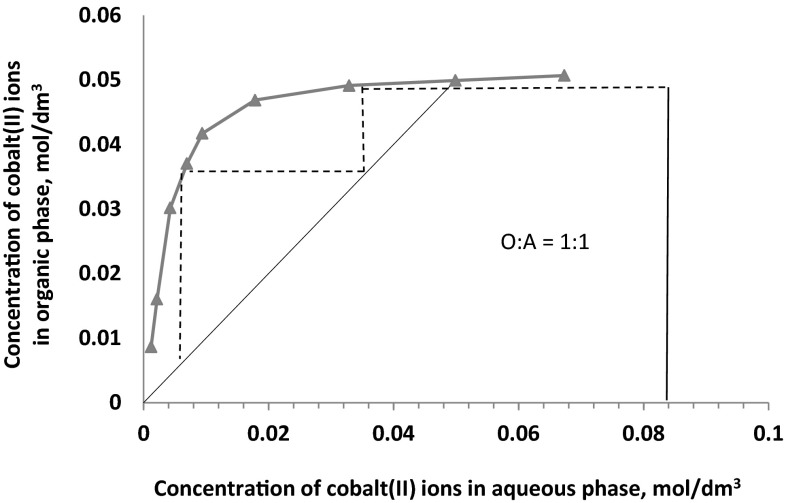
McCabe–Thiele plot for cobalt extraction from aqueous feed solutions containing 1 mol/dm^3^ Cl^−^, 0.5 mol/dm^3^ ${\text{SO}}_{4}^{2-}$ (phases ratio O:A **=** 1:1)

**Fig. 9:**
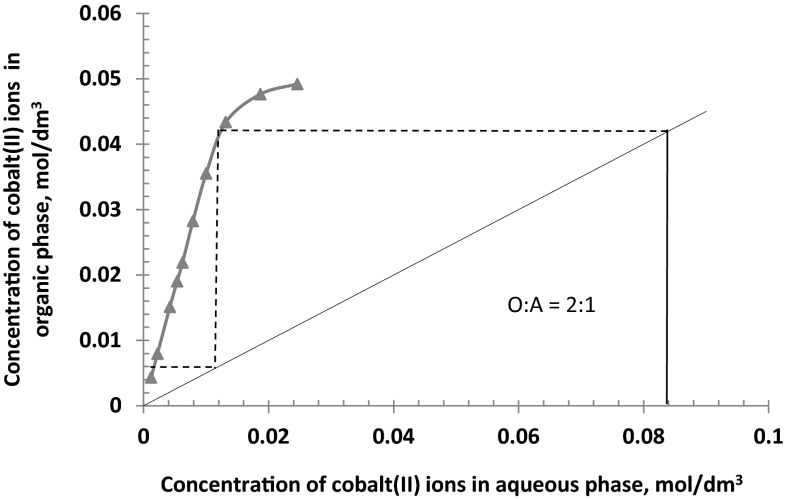
McCabe–Thiele plot for cobalt extraction from aqueous feed solutions containing 1 mol/dm^3^ Cl^−^, 0.5 mol/dm^3^ ${\text{SO}}_{4}^{2-}$ (phases ratio O:A **=** 2:1)

**Fig. 10:**
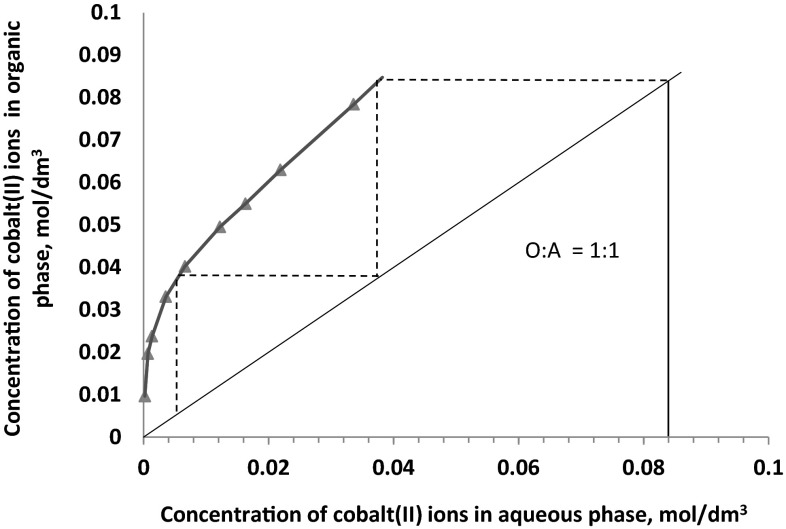
McCabe–Thiele plot for cobalt extraction from aqueous feed solutions containing 3 mol/dm^3^ Cl^−^, 0.5 mol/dm^3^ ${\text{SO}}_{4}^{2-}$ and at phases ratio O:A **=** 1:1

In this study the extraction isotherms have been obtained by contacting at O:A **=** 1 or 2 an aqueous solution containing 0.085 mol/dm^3^ Co(II), 0.5 mol/dm^3^${\text{SO}}_{4}^{2-}$ and 1 or 3 mol/dm^3^ Cl^−^ with 0.1 mol/dm^3^ toluene/dekan-1-ol solution of the 2-pyridineketoxime. The McCabe–Thiele diagrams presented in Figs. 8, 9 and 10 have shown that, regardless the chloride ions concentration and the phases ratio, the full cobalt(II) extraction can be achieved in two counter current stages. That predicted theoretical numbers of stages have also been confirmed by simulation tests and the assay results showed that from 1 M chloride solution cobalt(II) ions can be quantitatively extracted at the phases ratio of 1 in two counter current stages (94.8 % extraction) or in one stage at the phases ratio O:A = 2 (89 % extraction). In case of the 3 M aqueous solution, one stage counter current extraction process enables 76 % metal recovery, but in two-stages extraction cobalt(II) is transferred to the organic phase in 99.2 %.

### Stripping process

Our recent studies have indicated that 1-(2-pyridyl)tridecan-1-one oxime forms stable complexes with cobalt(II) ions especially in the presence of chloride ions and an effective re-extraction is possible at reducing conditions [19]. This knowledge enables to assume a comparably high stability of the complexes created in the studied chloride/sulphate mixture, therefore a stripping test has been carried out using an aqueous solution of HCl (10, 20 and 30 %) at 30 °C, at phase ratios of 1 and with addition or without a metallic reductant in either an inert atmosphere or air. The stripping test at inert conditions was carried out using argon as inert gas and in the first step of the experiment a loaded organic phase was first purged with argon to displace dissolved air. Next, 0.5 mmol of metallic cobalt as powder was inserted to the organic solution. The freshly prepared organic phases then were contacted for over 10 min with HCl solution. The results of the stripping experiments using a loaded organic solution containing 3.48 g Co^+2^/dm^3^ have been presented in the Table 1. The 20 % HCl in the presence of cobalt powder in argon atmosphere has been found to be the most effective stripping agent, which after 5 min of shaking at 30 °C strips 98.6 % of Co(II).

**Table 1:** Cobalt(II) stripping from loaded organic phase after extraction with 1-(2-pyridyl)tridecan-1-one oxime [initial concentration of Co(II) ions in the organic phase = 3.48 g/dm^3^]

Stripping agent	Stage of stripping	Co(II) concentration in stripping phase (mol/dm^3^)	Total recovery (%)
HCl 10 %, air	1	0.0001	0
	2	0.0001	
HCl 20 %, air	1	0.0007	2
	2	0.0005	
HCl 36 %, air	1	0.033	73
	2	0.010	
HCl 36 %, Ar, Co^0^	1	0.059	100
H_2_SO_4_ (from 5 to 95 %)	1	Emulsion	

### Mechanism of the complexation

2-Pyridineketoxime such as 1-(2-pyridyl)tridecan-1-one oxime can extract metal ions from the aqueous solutions through solvating mechanism or by forming a stable chelate, however, so specific organic extractant in many cases cannot form a clear one-type complex e.g. copper(II) ions are coordinated simultaneously by deprotonated oxygen of hydroxyimine group and by interaction with pyridine nitrogen. Similar solvating-chelate complexes seem to appear in case of cobalt(II) ions extraction: a coordination as a solvate enables extraction over a wide range of pH, but the coordination as a chelate (e.g. Co–O– bond results in a hydrogen cation release) enables a stable complex formation.

Based on these assumptions, the reactions of cobalt(II) ions complexation by 1-(2-pyridyl)tridecan-1-one oxime can be described as follows:

7 $$ {\text{Co}}_{\text{aq}}^{2 + } + \overline{{\left( {n + m} \right){\text{HL}}_{\text{org}} }} = \overline{{\left( {{\text{CoL}}_{n} \left( {\text{HL}} \right)_{{m, {\text{org}}}} } \right)^{(2 + ) - n} }} + n{\text{H}}_{\text{aq}}^{ + } $$

with

8 $$ K_{\text{ex}} = \frac{{\overline{{\left[ {\left( {{\text{CoL}}_{n} \left( {\text{HL}} \right)_{m} } \right)^{(2 + ) - n} } \right]}} \left[ {{\text{H}}^{ + } } \right]^{n} }}{{\left[ {{\text{Co}}^{2 + } } \right]\overline{{\left[ {\text{HL}} \right]}}^{(n + m)} }} $$

where

9 $$ \left[ {{\text{Co}}^{2 + } } \right] = {{\left[ {{\text{Co}}^{2 + } } \right]_{\text{T}} } \mathord{\left/ {\vphantom {{\left[ {{\text{Co}}^{2 + } } \right]_{\text{T}} } {\left( {1 + {\beta_{{{\text{SO}}_{4}^{2 - } }}} \left[ {{\text{SO}}_{4}^{2 - } } \right] + \mathop \sum \limits_{i = 1}^{4} \beta_{{i,{\text{Cl}}^{ - } }} \left[ {{\text{Cl}}^{ - } } \right]^{i} } \right)}}} \right. \kern-0pt} {\left( {1 + \beta_{{{\text{SO}}_{4}^{2 - } }} \left[ {{\text{SO}}_{4}^{2 - } } \right] + \mathop \sum \limits_{i = 1}^{4} \beta_{{i,{\text{Cl}}^{ - } }} \left[ {{\text{Cl}}^{ - } } \right]^{i} } \right)}} = {{\left[ {{\text{Co}}^{2 + } } \right]_{\text{T}} } \mathord{\left/ {\vphantom {{\left[ {{\text{Co}}^{2 + } } \right]_{\text{T}} } {\left( {1 + \beta_{{{\text{SO}}_{4}^{2 - } }} \left[ {{\text{SO}}_{4}^{2 - } } \right] + \beta_{{1,{\text{Cl}}^{ - } }} \left[ {{\text{Cl}}^{ - } } \right] + \beta_{{2,{\text{Cl}}^{ - } }} \left[ {{\text{Cl}}^{ - } } \right]^{2} + \beta_{{3,{\text{Cl}}^{ - } }} \left[ {{\text{Cl}}^{ - } } \right]^{3} + \beta_{{4,{\text{Cl}}^{ - } }} \left[ {{\text{Cl}}^{ - } } \right]^{4} } \right)}}} \right. \kern-0pt} {\left( {1 + \beta_{{{\text{SO}}_{4}^{2 - } }} \left[ {{\text{SO}}_{4}^{2 - } } \right] + \beta_{{1,{\text{Cl}}^{ - } }} \left[ {{\text{Cl}}^{ - } } \right] + \beta_{{2,{\text{Cl}}^{ - } }} \left[ {{\text{Cl}}^{ - } } \right]^{2} + \beta_{{3,{\text{Cl}}^{ - } }} \left[ {{\text{Cl}}^{ - } } \right]^{3} + \beta_{{4,{\text{Cl}}^{ - } }} \left[ {{\text{Cl}}^{ - } } \right]^{4} } \right)}} $$

In this equation *β*_*i*,*x*_ is an overall stability constant of *i*th cobalt(II) chloro-complexes or overall stability constant of cobalt(II) sulphate-complex ($\beta_{{{\text{SO}}_{4}^{2 - } }}$) (Table 2).

**Table 2:** Complex reaction formation and equilibrium constants at 298 K and at *I* **=** 0 mol/dm^3^ [24, 25]

Reaction	Equilibrium constant	log*β*
${\text{Co}}^{2 + } + {\text{Cl}}^{ - } = {\text{CoCl}}^{ + }$	$\beta_{{1,{\text{Cl}}}} = \frac{{\left[ {{\text{CoCl}}^{ + } } \right]}}{{\left[ {{\text{Co}}^{2 + } } \right]\left[ {{\text{Cl}}^{ - } } \right]}}$	−1.05
${\text{Co}}^{2 + } + 2{\text{Cl}}^{ - } = {\text{CoCl}}_{2}$	$\beta_{{2,{\text{Cl}}}} = \frac{{\left[ {{\text{CoCl}}_{2} } \right]}}{{\left[ {\left[ {{\text{Co}}^{2 + } } \right]} \right]\left[ {{\text{Cl}}^{ - } } \right]^{2} }}$	−3.75
${\text{Co}}^{2 + } + 3{\text{Cl}}^{ - } = {\text{CoCl}}_{3}^{ - }$	$\beta_{{3,{\text{Cl}}}} = \frac{{\left[ {{\text{CoCl}}_{3}^{ - } } \right]}}{{\left[ {{\text{Co}}^{2 + } } \right]\left[ {{\text{Cl}}^{ - } } \right]^{3} }}$	−5.28
${\text{Co}}^{2 + } + 4{\text{Cl}}^{ - } = {\text{CoCl}}_{4}^{ - 2}$	$\beta_{{4,{\text{Cl}}}} = \frac{{\left[ {{\text{CoCl}}_{4}^{ - 2} } \right]}}{{\left[ {{\text{Co}}^{2 + } } \right]\left[ {{\text{Cl}}^{ - } } \right]^{4} }}$	−6.62
${\text{Co}}^{2 + } + {\text{SO}}_{4}^{2 - } = {\text{Co}}({\text{SO}}_{4} )$	$\beta_{{{\text{SO}}_{4}^{2 - } }} = \frac{{[{\text{Co}}({\text{SO}}_{4} )]}}{{[{\text{Co}}^{2 + } ][{\text{SO}}_{4}^{2 - } ]}}$	2.47
${\text{H}}^{ + } + {\text{SO}}_{4}^{2 - } = {\text{HSO}}_{4}^{ - }$	$\beta_{{{\text{HSO}}_{4}^{ - } }} = \frac{{\left[ {{\text{HSO}}_{4}^{ - } } \right]}}{{\left[ {\left[ {{\text{H}}^{ + } } \right]} \right]\left[ {{\text{SO}}_{4}^{2 - } } \right]^{2} }}$	1.12
${\text{H}}_{2} {\text{O}} = {\text{OH}}^{ - } + {\text{H}}^{ + }$	${\text{pH}} = - \log (\gamma_{{{\text{H}}^{ + } }} [{\text{H}}^{ + } ])$	–

Taking account of the cobalt(II) ions complexation in the aqueous phase, the distribution ratio can be rewritten as follow:

10 $$ D = \frac{{\overline{{\left[ {\left( {{\text{CoL}}_{n} \left( {\text{HL}} \right)_{m} } \right)^{(2 + ) - n} } \right]}} }}{{\left[ {{\text{Co}}^{ + 2} } \right]_{\text{T}} }} = \frac{{K_{\text{ex}} \overline{{\left[ {\text{HL}} \right]}}^{{\left( {n + m} \right)}} }}{{\left[ {{\text{H}}^{ + } } \right]^{n} \left( {1 + \beta_{{{\text{SO}}_{4}^{2 - } }} \left[ {{\text{SO}}_{4}^{2 - } } \right] + \mathop \sum \nolimits_{i = 1}^{4} \beta_{{i,{\text{Cl}}^{ - } }} \left[ {{\text{Cl}}^{ - } } \right]^{i} } \right)}} $$

and

11 $$ { \log }D = { \log }K_{\text{ex}} + (n + m){ \log }\overline{{\left[ {\text{HL}} \right]}} - n{ \log }\left[ {{\text{H}}^{ + } } \right] + { \log }\left( {\frac{1}{{1 + \beta_{{{\text{SO}}_{4}^{2 - } }} \left[ {{\text{SO}}_{4}^{2 - } } \right] + \mathop \sum \nolimits_{i = 1}^{4} \beta_{{i,{\text{Cl}}^{ - } }} \left[ {{\text{Cl}}^{ - } } \right]^{i} }}} \right) $$

or

12 $$ { \log }D + { \log }\left( {1 + \beta_{{{\text{SO}}_{4}^{2 - } }} \left[ {{\text{SO}}_{4}^{2 - } } \right] + \mathop \sum \limits_{i = 1}^{4} \beta_{{i,{\text{Cl}}^{ - } }} \left[ {{\text{Cl}}^{ - } } \right]^{i} } \right) = { \log }K_{ex} + (n + m){ \log }\overline{{\left[ {\text{HL}} \right]}} + n{\text{pH}} $$

On the basis of the above considerations, the analysis of extracted complexes stoichiometry can be carried out using a graphical method, wherein the experimental values of the distribution coefficients of cobalt(II) ions are presented as a function of equilibrium pH and ligand’s concentration.

**Fig. 11:**
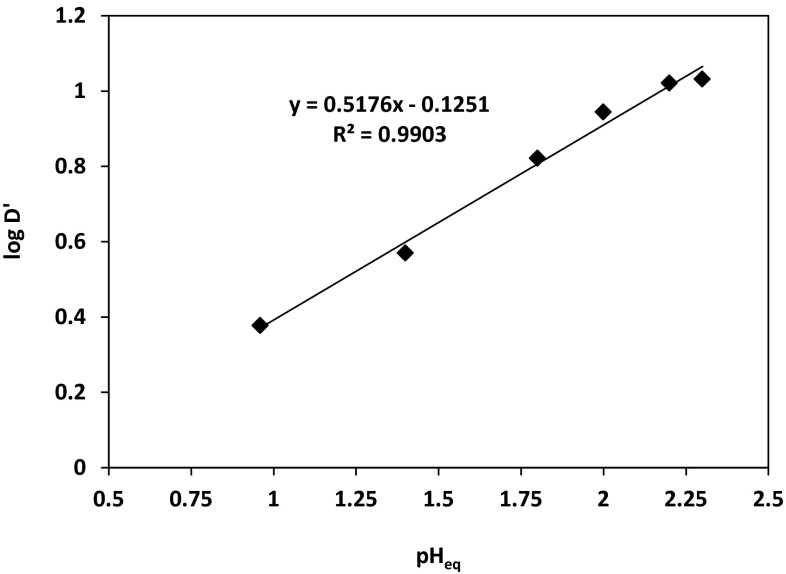
Logarithmic relation between Co(II) distribution coefficient $\left( {{ \log }D^{\prime} = { \log }D + { \log }\left( {1 + \beta_{{{\text{SO}}_{4}^{2 - } }} \left[ {{\text{SO}}_{4}^{2 - } } \right] + \mathop \sum \nolimits_{i = 1}^{4} \beta_{{i,{\text{Cl}}^{ - } }} \left[ {{\text{Cl}}^{ - } } \right]^{i} } \right)} \right)$ and equilibrium pH {[Co^2+^] **=** 0.01 mol/dm^3^; [${\text{SO}}_{4}^{2-}$] **=** 0.5 mol/dm^3^; [Cl^−^] **=** 1 mol/dm^3^; pH = 5; organic diluent: toluene with 10% (v/v) addition of decan-1-ol}

**Fig. 12:**
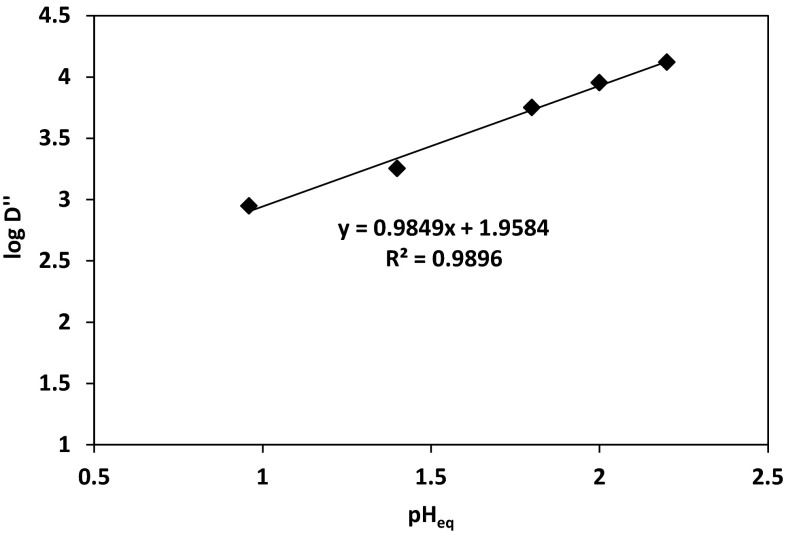
Logarithmic relation between Co(II) distribution coefficient $\left( {{ \log }\frac{{ [ {\text{Co]}}_{\text{org}} }}{{\left[ {\text{Co}} \right]_{\text{aq}}^{2} }} + { \log }\left( {1 + \beta_{{{\text{SO}}_{4}^{2 - } }} \left[ {{\text{SO}}_{4}^{2 - } } \right] + \mathop \sum \limits_{i = 1}^{4} \beta_{{i,{\text{Cl}}^{ - } }} \left[ {{\text{Cl}}^{ - } } \right]^{i} } \right)} \right)$ and equilibrium pH {[Co^2+^] = 0.01 mol/dm^3^; [${\text{SO}}_{4}^{2-}$] = 0.5 mol/dm^3^; [Cl^−^] = 1 mol/dm^3^; pH = 5; organic diluent: toluene with 10 % (v/v) addition of decan-1-ol}

Figure 11 illustrates the logarithmic plot of the cobalt(II) distribution coefficient $\left( {{ \log }D + { \log }\left( {1 + \beta_{{{\text{SO}}_{4}^{2 - } }} \left[ {{\text{SO}}_{4}^{2 - } } \right] + \mathop \sum \nolimits_{i = 1}^{4} \beta_{{i,{\text{Cl}}^{ - } }} \left[ {{\text{Cl}}^{ - } } \right]^{i} } \right)} \right)$ versus equilibrium pH. According the equation, the slope of the straight line given in the pH range between 1 and 3 should be close to 1 or its multiple, but it was found to be 0.52. That suggests that cobalt(II) ions coordination is a result of a binuclear complex formation stabilised by intramolecular hydrogen bond O–H–O [22, 23]. This theory could be confirmed by the logarithmic plot of the cobalt(II) distribution coefficient for the binuclear complex ($D = \frac{{ [ {\text{Co]}}_{\text{org}} }}{{\left[ {\text{Co}} \right]_{\text{aq}}^{2} }}$) versus equilibrium pH. The linear plot presented in Fig. 12 gives a slope equal to 0.985 (*R*^2^ **=** 0.990) which is in agreement that cobalt(II) ion complexation, as the binuclear complex resulted in one proton release to the aqueous phase. The protonation of the unbound ligand seems to be less possible in the studied range of pH, especially if the equilibrium pH decreases with the increase of the cobalt ions concentration in the organic phase.

**Fig. 13:**
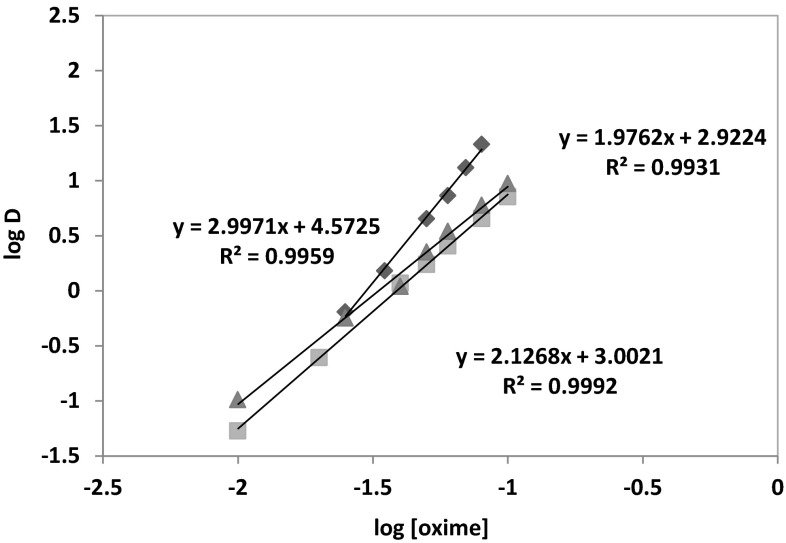
Logarithmic relation between Co(II) distribution coefficient and 1-(2-pyridyl)tridecan-1-one oxime concentration at different pH {pH **=** 0.5(* Filled black square*), 3(* Filled grey triangle*) or 5(* Filled grey square*); [Co^2+^] **=** 0.01 mol/dm^3^; [${\text{SO}}_{4}^{2-}$] **=** 0.5 mol/dm^3^; [Cl^−^] **=** 1 mol/dm^3^; pH **=** 5; organic diluent: toluene with 10 % (v/v) addition of decan-1-ol}

The graph $\left( {{ \log }D + { \log }\left( {1 + \beta_{{{\text{SO}}_{4}^{2 - } }} \left[ {{\text{SO}}_{4}^{2 - } } \right] + \mathop \sum \nolimits_{i = 1}^{4} \beta_{{i,{\text{Cl}}^{ - } }} \left[ {{\text{Cl}}^{ - } } \right]^{i} } \right)} \right)$ as a function of log[oxime] shows that at pH of 3.5 and 5 the slopes are close to 2 (1.976 and 2.127, respectively) thus suggesting a relationship of two molecules of the oxime with one molecule of Co(II) (Fig. 13).

A different stoichiometry has been observed in a strong acidic solution (pH **=** 0.5). This confirmed the formation of a different complex than in weak acidic conditions: the slope is equal to 2.997 and it suggests that three molecule of the oxime per cobalt molecule are involved in the coordination.

The role of the chloride ions concentration seems to correlate with cobalt-chloride species coordination, but looking at a speciation diagram (Fig. 4) a salts-out effect should mainly be taken into account. However, if chloride ions participate in the metal complexation, especially as balancer of complexes with positive charges, the reaction of the extraction can be expressed as:

13 $$ {\text{Co}}_{\text{aq}}^{2 + } + z{\text{Cl}}_{\text{aq}}^{ - } + \overline{{\left( {n + m} \right){\text{HL}}_{\text{org}} }} = \overline{{\left( {{\text{CoCl}}_{z} {\text{L}}_{n} \left( {\text{HL}} \right)_{{m, {\text{org}}}} } \right)^{{\left( {2 + } \right) - (n + z)}} }} + n{\text{H}}_{\text{aq}}^{ + } $$

with

14 $$ K_{\text{ex}} = \frac{{\overline{{\left[ {\left( {{\text{CoCl}}_{z} {\text{L}}_{n} \left( {\text{HL}} \right)_{m} } \right)^{{\left( {2 + } \right) - (n + z)}} } \right]}} \left[ {{\text{H}}^{ + } } \right]^{n} }}{{\left[ {{\text{Co}}^{2 + } } \right] \left[ {{\text{Cl}}^{ - } } \right]^{z} \overline{{\left[ {\text{HL}} \right]}}^{(n + m)} }} $$

therefore, the distribution ratio can be rewritten as follow:

15 $$ D = \frac{{\overline{{\left[ {\left( {{\text{CoCl}}_{z} {\text{L}}_{n} \left( {\text{HL}} \right)_{m} } \right)^{(2 + ) - n} } \right]}} }}{{\left[ {{\text{Co}}^{ + 2} } \right]_{\text{T}} }} = \frac{{K_{\text{ex}} \overline{{\left[ {\text{HL}} \right]}}^{{\left( {n + m} \right)}} \left[ {{\text{Cl}}^{ - } } \right]^{z} }}{{\left[ {{\text{H}}^{ + } } \right]^{n} \left( {1 + \beta_{{{\text{SO}}_{4}^{2 - } }} \left[ {{\text{SO}}_{4}^{2 - } } \right] + \mathop \sum \nolimits_{i = 1}^{4} \beta_{{i,{\text{Cl}}^{ - } }} \left[ {{\text{Cl}}^{ - } } \right]^{i} } \right)}} $$

and

16 $$ { \log }D + { \log }\left( {1 + \beta_{{{\text{SO}}_{4}^{2 - } }} \left[ {{\text{SO}}_{4}^{2 - } } \right] + \mathop \sum \limits_{i = 1}^{4} \beta_{{i,{\text{Cl}}^{ - } }} \left[ {{\text{Cl}}^{ - } } \right]^{i} } \right) = { \log }K_{\text{ex}} + z{ \log }\left[ {{\text{Cl}}^{ - } } \right] + (n + m){ \log }\overline{{\left[ {\text{HL}} \right]}} + n{\text{pH}} $$

but at different ionic strength the chloride ions concentration should be replaced by chloride ions activity $a_{{{\text{Cl}}^{ - } }} = \left[ {{\text{Cl}}^{ - } } \right]\gamma_{{{\text{Cl}}^{ - } }}$.

**Fig. 14:**
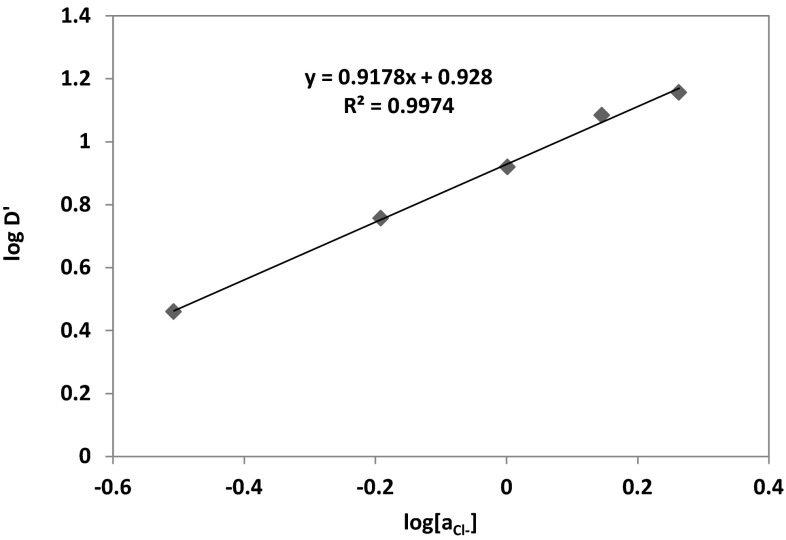
Logarithmic relation between Co(II) distribution coefficient $\left( {{ \log }D^{\prime} = { \log }D + { \log }\left( {1 + \beta_{{{\text{SO}}_{4}^{2 - } }} \left[ {{\text{SO}}_{4}^{2 - } } \right] + \mathop \sum \limits_{i = 1}^{4} \beta_{{i,{\text{Cl}}^{ - } }} \left[ {{\text{Cl}}^{ - } } \right]^{i} } \right)} \right)$ and chloride ions activity $\left( {a_{{{\text{Cl}}^{ - } }} } \right)$ {[Co^2+^] **=** 0.01 mol/dm^3^; [${\text{SO}}_{4}^{2-}$] **=** 0.5 mol/dm^3^; pH **=** 5; organic diluent: toluene with 10 % (v/v) addition of decan-1-ol}

According to above assumption a plot of ${ \log }D + { \log }\left( {1 + \beta_{{{\text{SO}}_{4}^{2 - } }} \left[ {{\text{SO}}_{4}^{2 - } } \right] + \mathop \sum \nolimits_{i = 1}^{4} \beta_{{i,{\text{Cl}}^{ - } }} \left[ {{\text{Cl}}^{ - } } \right]^{i} } \right)$ versus $\log a_{{{\text{Cl}}^{ - } }}$ constructed from experimental data obtained at constant initial pH and at constant cobalt and the oxime concentration should give a straight line with the slope indicating the number of chloride molecules participating in the cobalt complexation. The presented in Fig. 14 data give a slope equal to 0.9178 (*R*^2^ **=** 0.9974) and this means that cobalt(II) is probably coordinated by one molecule of the chloride. Thus, on the basis of the conducted graphical analysis, cobalt(II) ions at pH above 3 are transported into organic phase as a CoL(HL)Cl complex.

### Competitive extraction of Co(II) over Cu(II), Zn(II) and Ni(II)

**Fig. 15:**
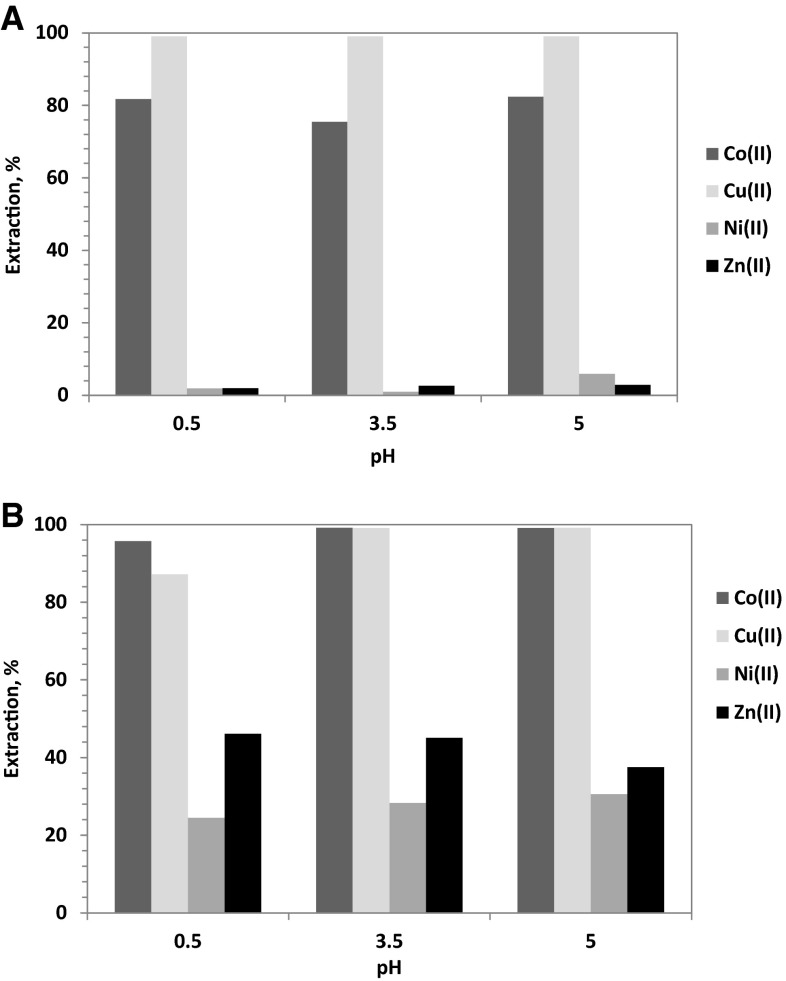
Influence of pH and chloride ions concentration on the extraction of Co(II), Cu(II), Ni(II) and Zn(II) from multielemental solutions with 1-(2-pyridyl)tridecan-1-one oxime dissolved in toluene with 10 % (v/v) addition of decan-1-ol {[oxime] **=** 0.1 mol/dm^3^; aqueous feed composition: 0.01 mol/dm^3^ Co(II), 0.01 mol/dm^3^ Cu(II), 0.01 mol/dm^3^ Ni(II) and 0.01 mol/dm^3^ Zn(II) at **a** 0.1 or **b** 5 mol/dm^3^ Cl^−^}

The competitive extraction of Co(II) from solutions containing 0.5 mol/dm^3^${\text{SO}}_{4}^{2-}$, 0.01 mol/dm^3^ Co(II), Cu(II), Zn(II) and Ni(II) ions and different concentration of chloride ions (0.1 or 3 mol/dm^3^ Cl^−^) was studied with 1-(2-pyridyl)tridecan-1-one oxime dissolved in toluene with 10 % addition of decan-1-ol. The obtained values of percent extraction has shown (Fig. 15a) that at low chloride ions concentrations (up to 0.1 mol/dm^3^) the studied oxime can be used for a selective extraction of Co(II) over Zn(II) and Ni(II) (extraction below 3 %), whereas the co-extracted Cu(II) can be selectively stripped with an aqueous solutions containing oxalate ions [16]. Unfortunately, the selectivity of the extraction process was reduced by increasing chloride ions concentration from 0.1 to 3 mol/dm^3^. As shown in Fig. 15b, at pH **=** 3.5 the percentage extraction of Zn(II) and Ni(II) increased from 2.6 to 45.1 %, whereas that of nickel(II) increased from 0.9 to 28.3 %, respectively. The co-extraction of the studied metals does not eliminate 1-(2-pyridyl)tridecan-1-one oxime as the extractant, but the selectivity of the stripping necessitates washing by water (Zn(II) re-extraction), next copper(II) should be stripped by mixing with 1 % solution of oxalic acid and 5 % Na_2_SO_4_, followed by nickel(II) stripping with 6–8 % HCl and remained in the organic phase Co(II) can be stripped with concentrated HCl in 90 %.

## Conclusion

The extraction of cobalt(II) from the chloride/sulphate aqueous solutions with the oxime of 1-(2-pyridyl)tridecan-1-one was studied as a function of pH, temperature, metal and chloride ions’ concentration in the aqueous feed, and concentration of the oxime in the organic phase.

The study showed a linear dependence of the cobalt extraction and the pH of the aqueous feed especially in the range of pH 1–3 (the extraction increased linearly from 70.5 to 91 %). The efficient extraction was also observed at strong acidic range of the pH, but during the extraction an emulsion was observed. Moreover, the influence of the chloride ions concentration on cobalt ions extraction indicated that the most visible effect was observed at range of Cl^−^ concentration 0–1 mol/dm^3^. Further increase of chloride ions concentration above 1 mol/dm^3^ Cl^−^ caused the process stabilisation and the recovery level was not lower than 90–92 %, moreover, the calculated enthalpy indicated that the extraction of Co(II) from the solutions containing above 1 mol/dm^3^ Cl^−^ was endothermic process.

The analysis of the cobalt concentration influence on the extraction also indicated the influence of chloride ions concentration on the loading capacity [0.5 and 0.78 mol of Co(II) per mol of the oxime at 1 and 3 mol/dm^3^ Cl^−^, respectively]. This effect may be attributed to the coordination of cobalt-chloride species which have a tendency to agglomeration especially at higher concentration of chloride solutions. Despite the high loading capacity, especially at the concentrated chloride solution, the McCabe–Thiele diagrams, confirmed by experimental data, show that regardless the chloride ions concentration and phases ratio, the full cobalt(II) extraction can be achieved in two counter current stages.

The stoichiometry studies of the complexes formed between cobalt(II) and oxime of 1-(2-pyridyl)trdecan-1-one indicated that cobalt(II) ions extraction underwent the hydrogen exchange reaction and chloride ions also participated in the complexes structure. Therefore, on the basis of the conducted graphical analysis, it was assumed that cobalt(II) ions at pH above 3 were transported into organic phase as a complex CoL(HL)Cl. The separation of Co(II) from Zn(II), Ni(II) and Cu(II) was also studied, but the selective recovery of the metals was possible using the multi-stage stripping process wherein the first stage was washing with water [Zn(II) re-extraction], next copper(II) was stripped by mixing with 1 % solution of oxalic acid and 5 % Na_2_SO_4_, the third stage was nickel(II) stripping with 6–8 % HCl and remained in the organic phase Co(II) was stripped with concentrated HCl in 90 %.
